# Ultraviolet C Decontamination Devices in a Hospital Pharmacy: An Evaluation of Their Contribution

**DOI:** 10.3390/pharmacy13010009

**Published:** 2025-01-25

**Authors:** Clara Baudart, Thomas Briot

**Affiliations:** 1 Hospices Civils de Lyon, Groupement Hospitalier Nord, Pharmacy Department, 69317 Lyon, France; clara.baudart@chu-lyon.fr; 2 LAGEPP, CNRS UMR5007, Université Claude Bernard Lyon 1, 69622 Villeurbanne, France

**Keywords:** microbial decontamination, UVC, drug compounding, disinfection methods, aseptic preparations, risk management

## Abstract

Purpose: The COVID-19 pandemic led to a major interest in ultraviolet C (UVC) disinfection devices and accelerated the implementation of UVC devices in healthcare facilities due to their proven efficacy in the inactivation of various pathogens. While UVC technology offers several advantages, some drawbacks remain. This report, drawing on studies, guidelines, and practical experiences related to the use of UVC technology in healthcare settings, examines the efficacy, advantages, and drawbacks of UVC devices, and their applications in aseptic drug-compounding pharmaceutical units. Summary: Studies, guidelines, and practical experiences were selected. UVC technology offers advantages such as rapid disinfection, reduced reliance on chemical agents, minimal waste, and freedom from manual disinfection variability, making it particularly valuable for maintaining aseptic conditions in compounding environments. However, some drawbacks persist, as it is a germ-dependent method and there is currently no standardized method for ensuring effectiveness. Conclusions: This opinion paper highlights the effectiveness of UCV technology in pharmaceutical compounding units, proving that it is a viable alternative to the traditionally used manual and operator-dependent methods. However, there is a need for standardized methods to evaluate UVC devices.

## 1. Introduction

Among the vast group of electromagnetic waves, light is composed of photons and the waves they create are classified according to their length. Ultraviolet (UV) waves range from 100 to 400 nm, and can be subdivided into three distinct areas comprising UVC (from 100 to 290 nm), UVB (from 290 to 320 nm), and UVA (from 320 to 400 nm). UVC is the most energetic type of radiation, and proteins as well as nucleic acids (RNA and DNA) absorb high quantities of UVC photons (a maximum of 280 and 260 nm, respectively) [1]. When interacting with DNA, the particularly energetic UVC allows its denaturation; thus, these waves are extremely harmful to human skin, but are also very interesting for eliminating pathogens [2,3,4,5].

It was reported that environments and surfaces in hospitals are often not properly disinfected [6] and that several bacteria (such as *Clostridium difficile*, methicillin-resistant *Staphylococcus aureus* (MRSA), vancomycin-resistant enterococci (VRE), *Acinetobacter baumannii*, or *Pseudomonas aeruginosa*) were able to survive on dry surfaces [7]. In pharmaceutical compounding units, and more specifically in cleanrooms, it was found that the operator is the main cause of microbial contamination [8]. To disinfect surfaces or vials in cleanrooms, alcohol wipes or other chemical products (such as quaternary ammonium, non-ionic surfactant, or hypochlorite), or impregnated wipes are often used by operators, and this cleaning method is the minimum standard mandatory in the US, according to the USP <797> [9]. This method is thus operator-dependent and there is potential for contaminants to remain on surfaces or medical devices [10,11]. To increase the robustness of the disinfection method, new automated tools have been developed, which have proven their efficacy in conjunction with a removal of visible soiling [12]. For instance, hydrogen peroxide vapor decontamination systems are very useful in hospitals and, more specifically, in the pharmaceutical compounding process (i.e., aseptic handling), in which chemotherapies, radiopharmaceutical preparations, or parenteral nutrition sources are prepared, for example [13,14,15]. This technology requires a complex installation process to ensure that there is no interaction between the air, workers, and adjacent rooms. On the other hand, the use of UVC in healthcare facilities is also increasing and is already spreading to many areas, such as operating theaters or hospital rooms [12]. In pharmaceutical compounding units, it can represent an alternative to the wiping and chemical automates described above by overcoming some of their drawbacks.

The objective of this report was to describe UVC technologies and their applications in healthcare settings, examine the efficacy, advantages, as well as drawbacks of UVC devices, and consider their applications in aseptic drug-compounding pharmaceutical units. To this end, an overview of the existing literature up to December 2023 was performed by all authors via a search of publications available on PubMed and Google Scholar.

## 2. Technologies to Produce UVC

UVC can be generated through several lamp technologies. Among them, low-pressure mercury lamps are the most commonly used [16,17]. They contain a small quantity of mercury vapor and emit two distinct UVC wavelengths: 253.7 nm and 184 nm. Medium-pressure mercury lamps emit a broader UV spectrum of UVC light but are less commonly used in germicidal applications. Excimer lamps produce intense UVC pulses by exciting noble gas (i.e., argon, krypton, or xenon) which then interacts with a halogen gas, creating an unstable molecule that is prone to emitting UVC light. More recently, light-emitting diodes (LEDs) have seen increasing use due to their many advantages. They can emit light at selected wavelengths or at various wavelengths simultaneously, which allows different LED wavelengths to be combined to deliver a synergistic effect for bacterial inactivation [16,18]. Another source of UVC is pulsed xenon lamps [17]. They produce light by passing electricity through ionized xenon gas. Approximately every six seconds, they flash germicidal UVC for a few milliseconds. These short bursts of UVC are combined with the thermal breakdown of cell walls through photon scattering, creating an effective germicidal process. Each technology has its own characteristics, advantages, and drawbacks (Table 1).

The choice of UVC lamp depends on the specific use of UVC, such as the required UVC intensity, wavelength, and operating conditions. Low-pressure mercury lamps remain the standard for many germicidal applications due to their efficiency, their long lifetime, and their produced wavelength, which is closer to maximum UVC germicidal irradiation (UVGI) [16,19]. UVC LEDs are seeing increasing use in portable and small-scale applications due to their compactness and energy efficiency [18].

## 3. Advantages of UVC

UVC decontamination systems have been used for many years in healthcare facilities, but also in cleanrooms disinfection, in water disinfection [20], in polluted wastewaters decontamination [21], and in food packaging sterilization [22]. UVC is a non-contact decontamination method with advantages and drawbacks compared to alternative decontamination strategies (Table 2). Its disinfecting action depends on the device’s properties (Table 1), the environment, and the pathogen in need of inactivation. Notably, UVC acts on bacteria, viruses, and fungi, which makes it a particularly effective decontamination device for ambient contamination. Many studies reported on the efficacy of UVC in SARS-CoV-2 inactivation [23,24,25] during the pandemic, and it is now widely used in healthcare facilities, operating rooms, and industrial production areas.

When devices are specifically designed for decontamination and are properly used, UVC can become the method of choice for achieving decontamination in aseptic compounding units. It is very easy to use and does not require its operators to undergo specific training, conversely to the traditional wiping method, where procedures have to be precise and rigorous [12,26]. Since UVGI is an automated method, it is a robust and repeatable method that is not influenced by the skills and behaviors of the operator. Moreover, since the disinfection process is automated, it saves workers time.

UVC is also a method of choice due to its high efficacy in surface microbial decontamination; Bruscolini et al. evaluated the efficacy of UVGI in the decontamination of a robot designed to produce chemotherapies (APOTECAchemo^®^). The authors reported an effective sterilization of the workplace inside the robot, even for very resistant microorganisms and when a high microbial charge was observed [27].

Notably, UVC is also presented as an environmentally friendly method [28]; waste management is a major issue in healthcare facilities [29]. A transition from wipes and chemical disinfection to UVC disinfection is supposed to inherently decrease the volume of waste and chemical products used. However, detailed data on the specific reduction in quantity must be considered according to energy consumption.

A main aspect of UVC technology is its non-contact method of decontamination. Due to this advantage, UVC boxes allow rapid disinfection of medical devices packaged in paper blisters, which would be altered by alcohol or other chemical vaporized liquid solutions. Before a sterile medical device (such as syringes, bags, or gloves) can enter a white room, the primary packaging (i.e., not sterile) has to be disinfected [9,26]. A common method involves a peroxide disinfection cycle lasting at least 70 min; this requires a sealed room or space to prevent vapor from spreading. In addition, an adequate aeration system is necessary to obtain non-toxic levels [30,31]. However, this method is not feasible in urgent situations, since this process is time-consuming and because the vapors produced are toxic to human health. UVC is therefore much faster and has a strong germicidal effect against several bacteria (vegetative and endospores) and viruses in few seconds (Table 2) [5]. Additionally, UVC systems have demonstrated high efficacy in inactivating aerosolized viruses, including coronaviruses. For example, 13.9 mJ/cm^2^ achieved a 2.2 log reduction, while higher doses of 49.6 mJ/cm^2^ resulted in a more than 3.7 log reduction (99.98% efficacy) in less than a minute, highlighting their rapid action and suitability for air purification applications [23]. Moreover, UVC devices are generally compact and easy to handle [32]. The dimensions of a UVC box can be easily adapted depending on the user’s needs, the available space, and the size of products or materials being disinfected. In pharmaceutical applications, a UVC box of around 1 m^3^ in size should be sufficient for disinfecting materials before they enter the white room. For this range of dimension, a qualified UVC device for medical application costs approximately EUR 10–15 k. Compared to a peroxide disinfection system, the installation of a UVC device is thus around 10 times less expensive. Finally, lamps are usually long-lasting, the maintenance requirements of UVC devices are relatively low, and they do not require any consumables, which further reduces the use-related cost over time, compared to that of other disinfection systems [16].

**Table 2:** Comparison of three microbial decontamination methods used in hospital pharmaceutical compounding units.

	UVC	Manual Disinfections	Peroxide Hydrogen Vapor Systems
Consumables required	No (✓)	Yes (✕)	Yes (✕)
Wastes generated	No (✓)	Yes (✕)	Yes (✕)
Cost of the method	++	+	+++
Time required for a reduction of 4 log_10_ CFU of *Clostridium difficile*	<20 min (2208 mJ.cm^−2^) [33]	20 min (0.5% sodium hypochlorite solution) >15 min (wipes impregnated with 0.07% peracetic acid, hydrogen peroxide, and acetic acid) [34].	60 min [34]
Time required for a reduction of 5 log_10_ of *Staphylococcus aureus*	<1 min (4 mJ.cm^−2^) [35]	5 s with 1.0% hydrogen Peroxide-impregnated wipes [36]	40 min (a dwell period is then necessary) [30]

(✓) advantage, (✕) drawback, (+) low, (++) moderate, (+++) high.

## 4. Drawbacks of UVC

Although this technology seems to be promising, especially in pharmaceutical compounding units, some drawbacks and limitations have to be raised (Table 3). First of all, the dose delivered by the UVC source depends on the distance between the target and the source, the intensity of the lamp, and on the exposure period. UVC devices have to be designed, developed, and qualified to ensure a homogenous light diffusion. As already mentioned, some microorganisms are more resistant to UVC radiation than others. However, despite the growth of this technology, data in the literature are still limited. It may therefore be difficult to answer all the questions regarding the required doses of UVC light [37]. In comparison to viruses and fungi, in bacteria, lower doses are used; variability arises due to non-standardized dose measurement methods [38,39].

The efficacy of this non-contact decontamination system also depends on the equipment and materials involved, and it cannot be used on active pharmaceutical ingredients that could be degraded upon exposure [40]. As an example, UVC has been identified as a reliable degradation method to eliminate chemotherapy agents like melphalan, etoposide, prednisone, and gemcitabine in waste fluids [41]. A large number of drugs and solvents are sensitive to UVC, exhibiting photodegradation; thus, they cannot be disinfected via UVC boxes without further study, which should aim to identify any potential UVC transfer across the packaging (vials of glass, for example). Another important limitation of this technology is the non-diffusion process: all surfaces have to be directly exposed to the light in order to be successfully treated, and surfaces that are not exposed (i.e., those in a shadowed area) are thus not disinfected.

It is of note that microorganisms have repair mechanisms after UVC irradiation, such as photoreactivation or dark repair, in which dimers repair is performed using photoenzymes present in bacteria (e.g., photolyase) or damaged DNA nucleotides are replaced by undamaged DNA in the absence of light, respectively [42,43]. An interesting review emphasized the mechanisms of DNA damage and repair [44]. For instance, it was found that using low UVC doses on cyanobacteria colonies enhanced biomass production (below 18 mJ/cm^2^ during 24 h of exposure) [45]. This phenomenon can be explained by slight stimulation of photosynthesis and enzyme activity occurring at a low dose, and is a concern, especially when UVC is used for water disinfection purposes. Moreover, Dai et al. studied the resistance of fungi cultures (*Trichophyton rubrum*) to UVC by exposing such a culture to five different cycles of UVC (120 mJ/cm^2^). Although there was no difference regarding the inactivation rates among the five consecutive cycles, the authors highlighted that DNA mutations in microorganisms exposed to UVC may occur and spread to daughter cells, in the absence of a DNA repair mechanism [46]. Similarly, Alcantara-Diaz et al. evaluated the adaptation of *Escherichia coli* to 80 consecutive cycles of sub-lethal UVC inactivation at 254 nm and reported that bacterial adaptation to cyclic UVC inactivation may result from the selection of beneficial mutations in genes associated with DNA repair and replication [47]. This information is crucial for the effective management of UVGI, emphasizing the importance of using suitable equipment. Any inadequacy in exposure time or insufficient UVC power can lead to undesirable microbial growth and contamination, with potentially severe consequences.

## 5. Discussion

The present literature overview highlights that the use of UVC light technology is an attractive strategy for disinfection in the pharmaceutical field, since it is rapid, operator-independent, and chemical-free. It overcomes the issues related to manual disinfection, providing more reproducible and efficient decontamination, especially compared to wiping [10,11,26]. Moreover, from a toxicological point of view, wipes and other chemical disinfectant solutions containing chlorine can irritate the skin or mucous membranes upon prolonged contact [48,49]. Similarly, detergent disinfectants cause corrosion when applied to metals, and have potential co-carcinogenic properties leading to skin cancer. Therefore, for users and for the environment, UVC seems to represent a safer option [24].

In the field of non-contact decontamination used in aseptic drug-compounding pharmaceutical units, UVC technology is attractive thanks to its lower cost compared to hydrogen peroxide systems. Moreover, this technology is easy to use and can be adapted to users’ needs, which make it highly suitable for various spaces. Regarding continuous productions, where prescriptions are received during ongoing processes, protocols and labels of bags or syringes that are manipulated into cleaned rooms are usually not disinfected by chemical methods due to their operating time. UVC, a rapid disinfection method, can therefore be used in such cases. However, particular attention must be paid to shadowed areas, in which UVC’s access is limited, reducing the disinfection capacity of the UVC box. This limitation should be overcome by increasing the number of UVC bulbs and using (or developing) materials that allow UVC to diffuse or reflect. Despite its proven efficiency, it was recently reported that in specific conditions, where surfaces were not cleaned before the use of UVC, the efficiency was altered [50]. Dirt and organic material present on surfaces or materials created shadowed areas that protected the microorganisms, which emphasizes the importance of performing mechanical cleaning before UVC disinfection, in accordance with the recommendations about UVC use. Further studies would allow more accuracy regarding the required UVC doses for each microorganism, as well as the exposure time required to achieve optimal disinfection. The evaluation of UVC device efficacy frequently relies on the European standard EN 14885, which was originally designed for chemical disinfectants [51,52]. This standard exclusively considers the reduction in the logarithmic (log10) count of microorganisms, without taking into account the potential repair mechanisms following bacterial inactivation. To complete standardization of disinfection methods, and to be more specific to UVC technologies, since 2022, BS 8628:2022, a British Standard, provides methods for quantitative testing of automated UVC device, which is a step towards a more standardized evaluation [53]. The comparison of UVC devices may be simplified by specifically focusing on evaluation methods to justify their bactericidal, mycobactericidal, sporicidal, yeasticidal, fungicidal, virucidal, or phagocidal activities. Prior to BS 8628:2022, only internal validations (performed by the users) could successfully qualify equipment, depending on the expressed needs of the users.

Finally, some questions persist regarding workers’ safety and, in particular, regarding ozone production and UVC exposure. Oxygen absorbs UV radiation below 200 nm, with a maximum absorption at 160 nm and ozone generation. An interesting review concluded that low-pressure mercury lamps manufactured with an appropriate envelope material cannot produce ozone, conversely to KrCl 222 nm lamps and quartz lamps that can be responsible for the production of ozone in the air [54]. Data are, however, reassuring, since the production of ozone is often below the limit of exposure (0.1 ppm for 8 h light work and 0.05 for 8 h heavy work), whether the air in the room is well-ventilated or whether the user stands far away from the UVC source [16]. Considering a UVC box designed for disinfection of materials during drug compounding, ozone production should not raise significant concerns, since the box is not confined to the production room. Moreover, workers will not be in close proximity to the box during their routine, and cleanrooms must be ventilated with high air renewal rates. It is, moreover, worth noting that ozone plays a beneficial role in enhancing the disinfection process, as reported in studies involving the combined use of UVC air irradiation and ozone treatment [55]. Regarding UVC exposure, many studies warned against the misuse of UVC devices during the COVID-19 pandemic, notably since UV is a mutagen, and the skin and eyes in particular have to be protected from UV exposure [56]. The International Electrotechnical Commission (IEC) and the Global Lighting Association even had to publish a position statement where they warned against UV exposure related to inadequate use or non-secure devices. For medical use, UVC medical devices are certified by the international standard ISO 15858 concerning UVC devices—permissible human exposure, allowing their safe and controlled use [57]. It defines the maximum exposure to UVC allowed (for example, 254 nm and 6 mJ/cm^2^ during an 8 h workday, as part of a 40 h work week) and the personal protective equipment or training required to ensure workers’ safety. Consequently, adequately qualified devices do not represent a risk for users, as the construction of the UVC box comprises specialized materials such as glass or metallic walls to shield users against UVC radiation. Furthermore, in case of accidental opening during a disinfection cycle, a security mode is activated, automatically turning off all lamps to ensure safety for the individuals in the area.

## 6. Conclusions

According to all the data gathered herein, UVC stands out as a method of choice in the field of aseptic compounding, and will certainly be extensively developed, enhanced by the progress of automation and securing of aseptic preparation. The list of benefits and advantages is extensive, though some drawbacks persist. However, the potential for improvement is evident, particularly regarding the increasing prevalence of UVC technology. Finally, this disinfectant technology appears to be a complement to other technologies, such as autoclave sterilization or peroxide disinfection, in the specific field of pharmaceutical compounding.

## Figures and Tables

**Table 1:** Main characteristics, advantages, and drawbacks of the UVC-lamp technologies available.

	Low-Pressure Mercury Lamps	Medium-Pressure Mercury Lamps	LEDs	Pulsed Xenon Lamps	Excimer Lamps	
					KrCl	KrBr
*Lifetime (hours) (claimed by producers)*	6000–8000	6000–8000	10,000	3000	3000	3000
*Wavelength produced (nm)*	253.7 and 184	265 to 275	255 to 27	200 to 1000	222	207
*Pulsed or continuous*	Continuous	Continuous	Both	Pulsed	Continuous	
*Use*	Air, water, surfaces	Water	Surfaces, package	Surfaces	Surfaces	
*Advantages*	Long lifetime High resistance: lamps are made of quartz		Without mercury Adjustable intensity and wavelength On/off instantaneous	Without mercury High resistance: lamps are made of quartz Low human skin penetration No warm-up time Low heating		
*Drawbacks*	Presence of mercury Produce heat Slow warm-up time before full output is achieved Possible ozone production		Risk of UV-B emission	Higher cost Ozone production		

**Table 3:** Main advantages and drawbacks of UVC decontamination.

Advantages	Disadvantages
+ Easy to use + Compact and long-lasting equipment + Quick method + Operator-independent efficiency + No chemicals used + No waste generated + Safe and efficient when used correctly	− Germ-dependent − No detergent action − No standardized method to ensure effectiveness − Various devices on the market, some unsuitable − Further research needed with regard to microbial resistance − Direct contact between surfaces and UVC is required

+ advantage, − drawback.

## Data Availability

No new data were created or analyzed in this study. Data sharing is not applicable to this article.
